# Antiphospholipid syndrome onset with hemolytic anemia and accompanied cardiocerebral events: a case report

**DOI:** 10.3389/fped.2024.1370285

**Published:** 2024-10-18

**Authors:** Jie Zheng, Zhao-Yu Wei, Shi-Chao Lin, Yong Wang, Xin Fang

**Affiliations:** Department of Pediatrics, FuJian Medical University Union Hospital, Fuzhou, China

**Keywords:** antiphospholipid syndrome, hemolytic anemia, neurological manifestations, myocardial infarction, case report

## Abstract

**Background:**

Antiphospholipid syndrome (APS) is a systemic autoimmune disorder that can manifest as thrombosis in the pediatric population, characterized by persistently positive antiphospholipid antibodies. APS is infrequently observed in children and could represent non-criteria manifestations.

**Case presentation:**

A six-year-old Chinese female presented with jaundice and dark urine, leading to a diagnosis of hemolytic anemia. Prednisone therapy initially improved her complexion, but she later developed neurological symptoms. Further laboratory tests showed intravascular hemolysis, coagulation abnormalities, and a positive lupus anticoagulant (LA) test result. Magnetic resonance imaging (MRI) scan revealed abnormal signals in the pons and cerebellar hemispheres, and an occluded part of the basilar artery. She was subsequently diagnosed with autoimmune encephalitis and received IG(immunoglobulin) and high-dose glucocorticoid (GC) treatment, leading to improvement in her clinical symptoms. However, the symptoms of hemolytic anemia worsened after two years. Subsequent laboratory assessments demonstrated the presence of intravascular hemolysis, coagulation abnormalities, and positive tests of anticardiolipin, LA, and anti-beta2 glycoprotein I antibodies. Elevated troponin I and N-terminal pro-brain natriuretic peptide levels, along with electrocardiogram and echocardiogram findings, indicated a myocardial infarction and a thrombus-like mass in the left auricle. Brain MRI showed multifocal infarction and cerebrovascular obstruction. She was diagnosed with APS accompanied by hemolytic anemia, cerebrovascular obstruction, and myocardial infarction. After several weeks of treatment with GC, IG, rituximab, hydroxychloroquine alone with low-molecular-weight heparin sodium, and warfarin, there was a marked improvement in the patient's condition.

**Conclusion:**

Pediatricians should be familiar with various presentations of pediatric APS to promptly detect possible aPL-related complications and initiate appropriate management strategies early on.

## Background

Antiphospholipid syndrome (APS) is a systemic autoimmune disorder characterized by an increased risk of thrombosis or pregnancy complications. Currently, there is no universally accepted set of criteria for pediatric APS, so the classification criteria established for adult-onset APS are often utilized for pediatric patients as well. The updated Sapporo criteria, currently the most widely utilized standard, mandates the existence of at least one clinical event (confirmed thrombosis in arteries, veins, or small vessels and/or pregnancy-related morbidity) and at least one persistently positive antiphospholipid antibody (aPL) test [lupus anticoagulant (LA), anticardiolipin (aCL), or anti-beta2 glycoprotein 1(aB2GP1)] for a formal diagnosis of APS (1). However, given the absence of pregnancy in the pediatric population, APS can only be diagnosed in instances of thrombosis, whether unprovoked, minimally provoked, or atypical. It's important to highlight that risk factors such as cigarette smoking, atherosclerosis, hypertension, and hyperlipidemia are more prevalent among adults. Therefore, non-thrombotic clinical manifestations such as thrombocytopenia, hemolytic anemia, and neurological disorders often precede overt thrombosis in children with APS (2). This implies that applying diagnostic criteria designed for adult-onset APS to pediatric cases might lead to underdiagnosing or delayed diagnosis in children.

The American College of Rheumatology (ACR) and the European League Against Rheumatism (EULAR) have collaboratively formulated the classification criteria for APS in 2023, which offer more comprehensive coverage of clinical domains, expanding beyond the Sapporo criteria to encompass cardiac valve damage and thrombocytopenia. While these revised criteria may enhance the identification of pediatric APS, it is essential to note that their specificity and sensitivity for the pediatric demographic remain to be empirically validated (3). This is due to the potential divergence in atypical manifestations between the pediatric and adult populations, let alone pediatric APS seems to be more severe (4). Furthermore, the optimal treatment for arterial thrombotic complications in patients with APS has not yet been fully established. Evidence on the use of anticoagulation for thromboembolism is primarily inferred from adult practice, and data on INR ranges and dose adjustments for children are also extrapolated from adult practice (5). Hence, it is crucial to summarize the clinical manifestations and clinical practice experiences in pediatric patients.

Herein, we report a rare case of a six-year-old female Chinese patient with hemolytic anemia and accompanied cardiocerebral events, who was eventually diagnosed with APS at eight years of age.

## Case presentation

In January 2021, a six-year-old Chinese girl with a yellowish tint to her skin and scleras and dark urine was diagnosed with hemolytic anemia. She had no past or family history of autoimmune diseases. Prednisone (PDN) treatment improved her complexion slightly, but her urine color remained dark. Later in June, she presented with paroxysmal vertigo several times a day, lasting for a few minutes per time. Ten days later, she exhibited unsteady walking, followed by slurred speech a few hours afterward, with occasional choking on liquids. She also experienced pain in her left toe and leg. A physical exam revealed red macules on her left foot and toe, and positive finger-nose and heel-to-toe tests. Bilateral Babinski signs were present.

**Figure 1:**
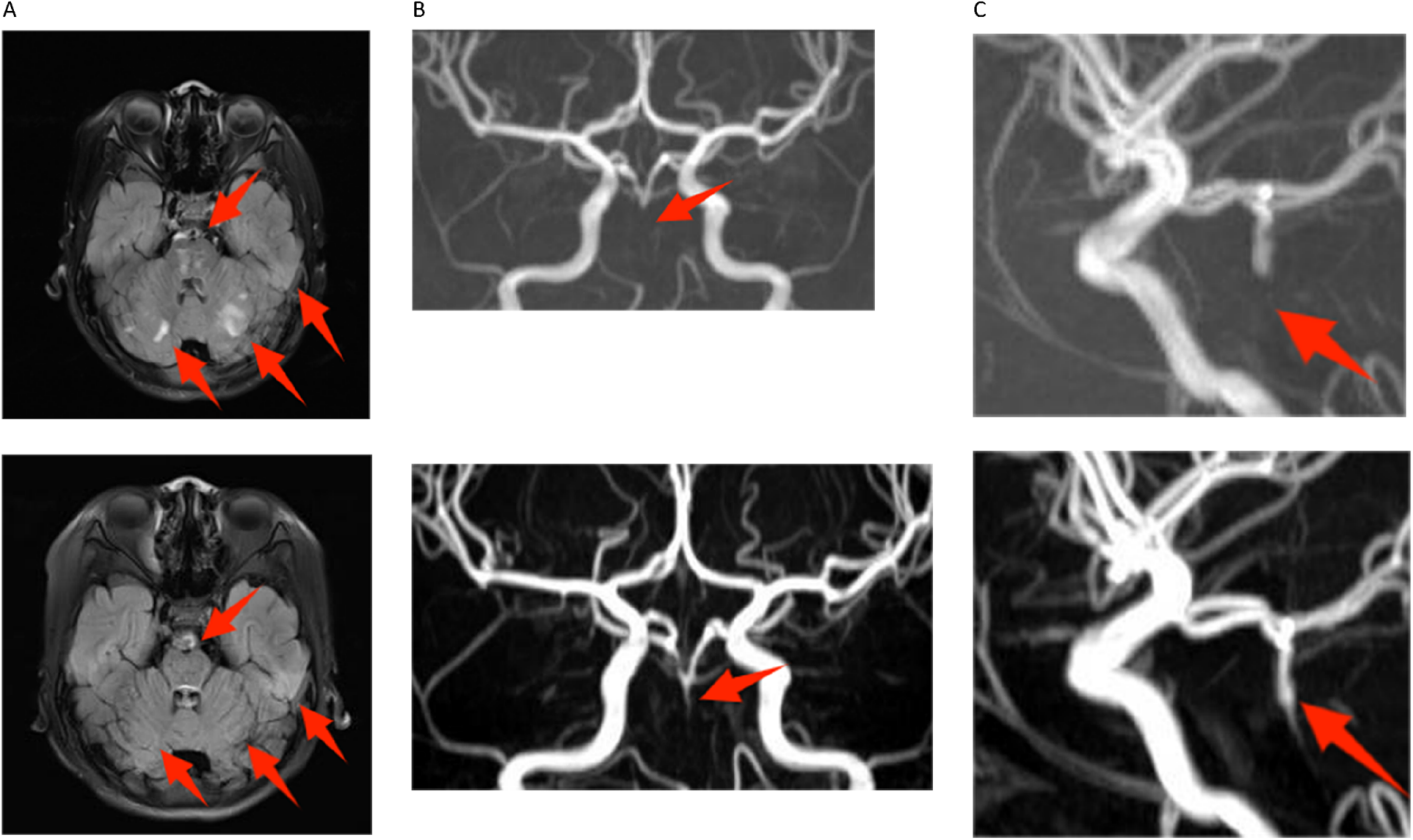
Magnetic resonance imaging in both hospitalizations. The image above is from 2021, and below is from 2023. **(A)** Compared to the 2021 image, the fluid-attenuated inversion recovery (FLAIR) images of transverse relaxation time (T2) weighted from 2023 showed multiple old and new abnormal signals in bilateral pons and cerebellar hemispheres. Notably, the size of the old lesion has decreased (indicated by red arrows). **(B**,**C)** The proximal part of the basilar artery was not visualized on digital subtraction angiography in either the 2021 or 2023 images. However, the middle and distal segments were partially visualized in the 2023 image (indicated by red arrows).

**Figure 2:**
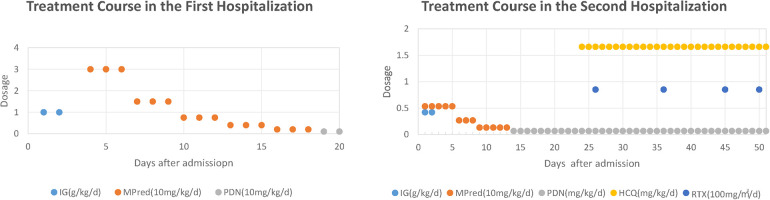
Treatment course in both hospitalizations. IG, immunoglobulin; mPDN, methylprednisolone; PDN, prednisone; HCQ, hydroxychloroquine; RTX, rituximab. Treatment Course in the First Hospitalization: IG at 1 g/kg/day from day 2 to day 3; mPDN at 30 mg/kg/day from day 4 to day 6, then 15 mg/kg/day from day 7 to day 9, and 7.5 mg/kg/day from day 10 to day 12, followed by 4 mg/kg/day from day 13 to day 15, then 2 mg/kg/day from day 16 to day 18; PDN at 1 mg/kg/day from day 19 to day 20. Treatment Course in the Second Hospitalization: IG at 0.42 g/kg/day from day 1 to day 2; mPDN at 5.33 mg/kg/day from day 1 to day 5, then 2.67 mg/kg/day from day 6 to day 8, and 1.33 mg/kg/day from day 9 to day 13; PDN at 0.67 mg/kg/day from day 14 to day 51; HCQ at 1.66 mg/kg/day from day 23 to day 51; RTX at 85 mg/m^2^/day administered on days 26, 36, 45, and 50.

Laboratory tests showed intravascular hemolysis with a negative direct Coombs test, significantly extended activated partial thromboplastin time (APTT), and a positive LA test result (Table 1). Magnetic resonance imaging (MRI) scan revealed abnormal signals in the pons and cerebellar hemispheres. Longitudinal relaxation time (T1) weighted imaging showed low signals and diffusion-weighted imaging showed partially high and isointense signals (Data not shown), transverse relaxation time(T2) weighted and fluid-attenuated inversion recovery (FLAIR) imaging showed high signals (Figure 1A). Magnetic resonance angiography (MRA) suggested part of the basilar artery was not visualized, which was considered a congenital variation at that time (Figures 1B,C). Based on these findings, the patient was diagnosed with autoimmune encephalitis. She received IG and methylprednisolone (mPDN) therapy (dose and duration of each treatment are detailed in Figure 2). Symptoms improved, and she was prescribed oral PDN (Figure 2). One month later, the MRI showed reduced inflammation (Data not shown). One year later in the second follow-up visit, laboratory tests were normal, except for a positive LA and slightly extended APTT (Table 1). The patient has not had any recurrence of symptoms in two years.

**Table 1:** Laboratory findings.

Variable	Normal range	First hospitalization	First follow-up visit	Second follow-up visit	Second hospitalization
		2021-06	2021-12	2022-06	2023-05
APTT(S)	(28–42)	77.8	84.1	67.2	79.5
FIB(g/L)	(4–2)	1.88	NA	2.94	6.16
INR	(0.8–1.5)	1.13	NA	0.93	1.23
WBC(10^9 ^/L)	(4–10)	8.70	12.31	9.80	14.69
HB(g/L)	(120–165)	108.0	136	113.0	48
PLT(10^9 ^/L1)	(100–300)	318	342	341	219
TBIL(umol/L)	(2–22)	35.8	NA	NA	107.2
IBIL(umol/L)	(0–20)	24.8	NA	NA	86.0
ANA		<1:100	NA	<1:100	1:320+
Coombs test		(−)	NA	NA	(+)
Antiphospholipid antibodies					
aCL^a^IgM(MPL/ml)	(0–18)	6.22	NA	NA	24.90
aCL^a^IgG(GPL/ml)	(0–18)	5.80	NA	NA	90.60
LA^b^	(0–1.2)	2.06	2.49	2.12	1.78
aB2GP1^c^(RU/ml)	(0–20)	NA	71.50	NA	NA
aB2GP1^a^IgM(AU/ml)	(16–24)	NA	NA	NA	156.00
aB2GP1^a^IgG(AU/ml)	(16–24)	NA	NA	NA	89.40

APTT, activated partial thromboplastin time; FIB, fibrinogen; INR, international normalized ratio; WBC, white blood cell count; HB, hemoglobin; TBIL, total bilirubin; IBIL, indirect bilirubin; ANA, antinuclear antibody; PLT, platelet; LA, lupus anticoagulant; aCL, anticardiolipin; Ig, immunoglobulin; aB2GP1, anti-beta2 glycoprotein 1.

^a^ Detected by chemiluminescence immunoassay.

^b^ Detected through Russell viper venom time.

^c^ Detected via enzyme-linked immunosorbent assay.

**Figure 3:**
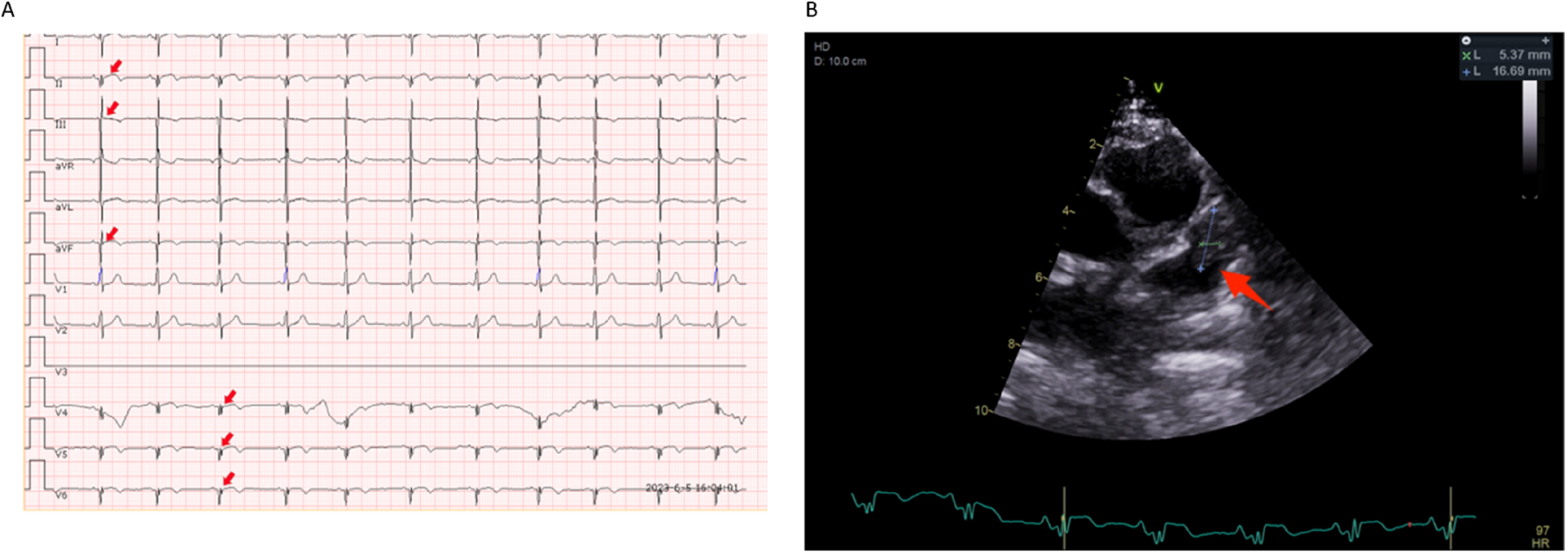
Electrocardiogram and echocardiography images in the second hospitalizations. **(A)** Sinus tachycardia and ST segment back elevation in II, III, aVF, and V4–V6 lead (indicated by red arrows). **(B)** A thrombus-like clump can be observed in the left auricle(indicated by blue lines and a red arrow).

Then the patient presented with a yellowish tint to her skin and scleras and tea-colored urine again. Subsequent laboratory assessments demonstrated elevated levels of troponin I of 2.240 μg/L (normal range: 0.00–0.02 μg/L) and N-terminal pro-brain natriuretic peptide of 5525 pg/ml (normal range: 0–115 pg/ml), along with a positive direct Coombs test. Additionally, laboratory findings indicated the presence of intravascular hemolysis, significantly extended APTT again, and a positive aPL test, encompassing aCL, LA, and aB2GP1 antibodies (Table 1). An electrocardiogram (ECG) showed sinus tachycardia and ST elevation in leads II, III, aVF, and V4–V6 (Figure 3A). A subsequent computed tomography angiography (CTA) test was conducted, revealing no evidence of embolism in the coronary arteries (Data not shown). Echocardiography (ECHO) revealed left ventricular wall motion abnormalities, a thrombus-like clot in the left auricle, decreased systolic function, and trace pericardial effusion (Figure 3B). MRI of the brain showed new abnormal signals in the right frontal and parietal lobes, with scattered signals in the brainstem and cerebellar hemispheres that had decreased in size. Compared to the previous MRA, the proximal part of the basilar artery was still not visualized, but the middle and distal segments were visualized, although were slender (Figures 1B,C). The original lesions in the cerebellum and pons have evolved into softening foci, indicating possible previous ischemic damage, suggestive of obstruction rather than congenital variation (Figure 1A).

The patient was diagnosed with APS accompanied by hemolytic anemia, cerebrovascular obstruction, and myocardial infarction (MI). After several weeks of treatment with glucocorticoid (GC), IG, rituximab (RTX), and hydroxychloroquine (HCQ) alone with simultaneous administration of warfarin and low-molecular-weight heparin sodium (Figure 2), the patient's skin and urine color returned to normal, indicating improvement in the condition. The ECG results demonstrated significant improvement, with only minor abnormalities in Q and T waves observed in leads II, III, aVF, and V5–V6, and ECHO confirmed the resolution of the blood clot (Data not shown). The patient's international normalized ratio (INR) was maintained within the target range of 2.0–3.0 (2), indicating effective anticoagulation therapy. The patient was discharged with a prescription for maintenance therapy with HCQ and PDN. Warfarin and low-molecular-weight heparin sodium were also prescribed to maintain the INR within the desired range. The patient is currently under close follow-up at the outpatient clinic and is well tolerated with the treatments according to the feedback.

## Discussion and conclusions

The onset of APS during childhood can occur at any age, typically between the ages of 9 and 14. Although the prevalence of thrombosis among aPL-positive patients is generally lower than among adults, pediatric APS is almost always associated with thrombosis (6), which can manifest as venous thrombosis(60%), arterial thrombosis(32%), small vessel thrombosis (6%), or a combination of arterial and venous thrombosis (2%) (7). While the proportion of arterial thrombosis cases is relatively small, the risk of recurrence is higher compared to venous thrombosis cases of APS (2).

It is also important to note that non-thrombotic manifestations are more frequent in children and may even precede thrombotic manifestations (8, 9). A recent cohort study of aPL-positive children showed 90% did not develop any thrombotic event while hematological manifestations were the most frequent (42%), followed by neurological (19.8%) manifestations (10). Similar results were obtained in pediatric APS patients. Hematologic disorders were observed in 38% of patients, making them the most frequently associated non-thrombotic manifestation (7). Neurological manifestations such as ischemic stroke represent the most commonly observed arterial thrombotic event among children (9), even more so than in adults (7). However, some neurologic manifestations may also be attributed to immune-mediated vascular, inflammatory, and direct neuronal effects (11, 12). As shown in Table 2, symptoms like hemiparesis, and altered consciousness could be found in patients with or without evidence of occlusion of cerebral vessels.

**Table 2:** Pediatric APS with neurological manifestations.

Reference	Year	Demographic features	Scenario	Complications	Management/outcome
Nordal et al. (13)	1999	12-year-old girl	Chorea, diffculty speaking. CT revealed decreased circulation in both basal ganglia and parts of the temporal lobes.	Primary APS	Anticoagulation, glucocorticoids, cyclophosphamide, azathioprine.
Shabana et al. (14)	2009	9-year-old Saudi girl	Recurrent attacks of delirium with drooling of saliva. Depressive with abnormal behaviour, hallucinations, drowsiness, confusion and increased hours of sleep.	Primary APS	Aspirin, hydroycloroquine, and antidepressants.
Freeman et al. (15)	2014	Case 1: 13-year-old girl	Weakness right arm and leg, recurrent episodes of involuntary movements of right shoulder, arm and hand. Intermittent episodes of the sensation of burning or pain in fingers and toes. Extreme fatigue and occasional dizziness.	Secondary to SLE	Anticoagulant, hydroxychloroquine. glucocorticoids, hycophenolatemofeti.
		Case 2: 9-year-old girl	Seizures (generalised tonic–clonic, left sided focal which secondarily generalised), left hemiparesis and slurred speech.	Primary APS	Anticoagulation therapy
Marzooq (16)	2023	2-year-old girl	Sudden right-sided hemiparesis and altered consciousness. CT revealed a massive left middle cerebral artery ischemic stroke.	Primary APS	Anticoagulation and antiplatelet therapy
Qinghua et al. (17)	2024	14-year-old girl	Cognitive impairment and chorea-like movements. MRI revealed a left-sided infarct in the midbrain.	Primary APS	Anticoagulation, glucocorticoids, intravenous immunoglobulin, and multiple plasma infusions.

CT, computed tomography; MRI, magnetic resonance imaging; SLE, systemic lupus erythematosus.

According to Lóczi et al., the majority of MI cases in APS patients initially manifest as MI, accounting for 73% of cases (2). These cases exhibit specific clinical characteristics, including a relatively young age at onset, absence of gender preponderance, predominantly without signs of atherosclerosis, and a heightened risk of recurrent thrombotic events. The literature on pediatric APS patients presenting with cardiac manifestations is scarce. Based on the limited number of pediatric case reports available (Table 3), the majority of cases involve primary APS, and there is no significant gender preponderance. It appears that MI in pediatric APS patients is more likely associated with thrombus formation in the coronary arteries as suggested in Table 3, although we have not found evidence of embolism in the coronary arteries in our case. The underlying pathophysiology of MI in APS is intricate and involves multiple factors. Other potential contributors to the development of MI include coronary spasm and spontaneous coronary dissection, apart from coronary thrombosis (2). Unlike our case where thrombosis was observed in the left atrium, two reported cases indicate thrombosis occurred in the right coronary artery, and one case in the left ventricle. The analysis may be prone to biases arising from the limited size of the available data. Therefore, it is imperative to acquire additional data, such as a larger cohort, to ensure a comprehensive and unbiased analysis of the clinical features.

**Table 3:** Pediatric APS with cardiac manifestations.

Reference	Published year	Demographic features	Scenario	Complications	Management/outcome
Miller et al. (18)	1995	8-year-old African-American girl	Transmural MI associated with thrombosis of coronary artery, small intramural arteries, and coronary arteriopathy.	Secondary to SLE	N/A
Clauss et al. (19)	2003	2-year-old American boy	A history of cardiogenic shock, severe mitral and tricuspid regurgitation and recurrent thrombosis.	Primary APS	N/A
Al-Kiyumi et al. (20)	2003	11-year-old Omani boy	Left ventricular thrombi and dilated cardiomyopathy.	Primary APS	Expired due to multiorgan failure.
Morchi et al. (21)	2009	17-year-old boy	Acute MI associated with thrombus in the right coronary artery.	Primary APS	Eptifibatide, heparin, and aspirin for 48 h, then started on warfarin.
Moreno-Ruiz et al. (22)	2010	14-year-old girl	MI (anterolateral and apical)	Primary APS	Warfarin, prednisone, and chloroquine
Waisayarat et al. (23)	2019	12-year-old Thai boy	Thrombosis in the right atrium.	Primary APS	Expired from heart failure.
Yan et al. (24)	2020	8-year-old Chinese girl	Thrombus in the right atrium.	Secondary to SLE, complicated with LN	Heparin for 2 weeks. Methylprednisolone for 3 days followed by oral steroids.

SLE, systemic lupus erythematosus; LN, lupus nephritis; MI, myocardial infarction; N/A, not available.

There are some other limitations in our presentation, such as the lack of detailed profiles at the onset of the disease and no full scale of her past and family histories. However, upon retrospective analysis, the patient initially exhibited autoimmune hemolytic anemia, albeit with aberrant blood coagulation test results, an atypical presentation in hemolytic anemia, which prompted consideration of a potential aPL-related etiology, and aPL panel could be a valuable diagnostic tool. Subsequently, the patient developed symptoms such as paroxysmal vertigo, gait instability, and dysarthria, which are indicative of the diverse neurological manifestations that can accompany APS and therefore warranted a comprehensive neuroimaging evaluation, like CTA or MRA, to rule out the presence of ischemic stroke or thrombotic events. Additionally, routine ECG and ECHO may aid in detecting subtle myocardial infarction or cardiovascular thrombosis. A comprehensive autoimmune panel would be useful in identifying secondary APS.

In conclusion, pediatricians should be well-versed in the diverse presentations of pediatric APS to promptly identify potential aPL-related complications and implement appropriate management strategies early on. Non-criteria manifestations should be carefully considered, along with factors such as the patient's aPL profile and other thrombotic or bleeding risks.

## Data Availability

The original contributions presented in the study are included in the article/supplementary material, further inquiries can be directed to the corresponding authors.
